# The role of STIM proteins in neutrophil functions

**DOI:** 10.1113/JP275639

**Published:** 2018-03-23

**Authors:** Nicolas Demaurex, Stephanie Saul

**Affiliations:** ^1^ Department of Cell Physiology and Metabolism University of Geneva Geneva 1211 Switzerland

**Keywords:** ion channels, phagocytosis, calcium signalling, store‐operated Ca^2+^ entry, NADPH oxidase, animal models

## Abstract

Stromal interaction molecule (STIM) proteins regulate store‐operated Ca^2+^ entry (SOCE) in innate and adaptive immune cells and participate in the Ca^2+^ signals that control the functions of neutrophils, the first line of host defence against bacterial and fungal infections. Loss‐of‐function experiments in animal and cellular models indicate that both STIM1 and STIM2 regulate neutrophil functions, but the complexity of the SOCE machinery and the versatility of neutrophils complicate the evaluation of the results. This review aims to summarize the latest progress in the field, with special attention to the details of the experimental designs. Future study design should aim to improve the standardization of experimental procedures and to provide a more holistic understanding of the role of STIM proteins in neutrophils function.

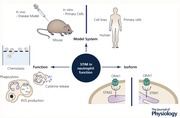

## Ca^2+^ signalling in neutrophil function

Neutrophils are highly versatile cells of the innate immune system and form the first line of host defence when recruited from the blood stream to sites of inflammation to target invading pathogens (Nauseef & Borregaard, 2014). Neutrophils’ antimicrobial responses include cytokine secretion, the release of granule components that target pathogens or shape the extracellular environment, the production of intra‐ and extracellular reactive oxygen and nitrogen species (ROS and RNS) that act as second messengers and participate in pathogen clearance, and the phagocytosis of pathogens (Nauseef & Borregaard, 2014; Rosales *et al*. 2016). Besides antimicrobial functions, neutrophils and the other granulocytes – basophils and eosinophils – are involved in auto‐immune and allergic responses, as an excessive recruitment and activation of these cells can cause severe tissue damage (Siracusa *et al*. 2013; Thieblemont *et al*. 2016). Early studies reported that intracellular Ca^2+^ signals control innate and adaptive immune cell function, including the recruitment and antimicrobial functions of neutrophils. The SOCE pathway mediated by STIM1‐ORAI1 interactions was then shown to be essential for T lymphocyte activation and proliferation and to regulate neutrophil functions (Clemens & Lowell, 2015). Understanding the regulation of neutrophil functions and the potential to fine‐tune these processes in a therapeutic context therefore requires a detailed understanding of the regulation of SOCE.

Within the last decade, significant progress has been made in understanding the SOCE machinery, and the dynamic STIM‐ORAI interactions have been clarified at the cellular and molecular level (Hogan *et al*. 2010; Berna‐Erro *et al*. 2012; Shaw *et al*. 2013). SOCE is activated by the generation of inositol trisphosphate (InsP_3_) following the engagement of plasma‐membrane receptors. InsP_3_ binds and opens its cognate receptors in the endoplasmic reticulum (ER), triggering a Ca^2+^ release from intracellular stores. The drop in ER‐luminal Ca^2+^ concentration leads to dissociation of Ca^2+^ from EF‐hands of STIM1 and STIM2 proteins residing in the ER membrane. Subsequently, STIM proteins undergo a conformational change and translocate to ER‐plasma‐membrane contact sites. Here they trap and gate Ca^2+^‐permeable channels of the ORAI and TRP families, of which several isoforms exist, enabling a sustained influx of Ca^2+^ into the cytosol.

STIM proteins are complex and multifaceted regulators of SOCE (Oh‐Hora *et al*. 2008; Soboloff *et al*. 2012). The discoveries of functional STIM1 and STIM2 splice variants (Darbellay *et al*. 2011; Miederer *et al*. 2015; Rana *et al*. 2015) and the identification of cysteine residues subject to oxidation (Hawkins *et al*. 2010) revealed that STIM‐mediated functions can be subject to complex physiological regulation, such as redox modulation and differential expression of protein variants (Bhardwaj *et al*. 2016; Niemeyer, 2016). The presence of two ORAI‐gating STIM isoforms allows a specialized regulation of physiological processes by a specific isoform. This extending knowledge demands a more detailed analysis of the different STIM variants and their regulation to fully understand their role in neutrophil function. We have recently reviewed the current knowledge on the role of SOCE in phagocytes (Demaurex & Nunes, 2016), and will focus here on recent studies on neutrophils. These studies confirm that STIM1 regulates neutrophil ROS production and phagocytosis, and indicate that STIM2 contributes to the transcriptional control of cytokine production. However, due to differences in experimental design, the role of STIM proteins in regulating chemotaxis and neutrophil recruitment remains unclear.

Recently, Clemens and colleagues published a comprehensive study using mice lacking STIM1, STIM2 or both proteins in the myeloid and neutrophil‐specific lineage. The study revealed a cooperative role of STIM1 and STIM2 in mediating SOCE and regulating cytokine production (Clemens *et al*. 2017). These findings confirm that STIM1 is the major regulator of SOCE and show that STIM2 contribution is limited to low doses of specific physiological agonists, with fMLF tested in this study as such. STIM1 was further shown to promote ROS production and phagocytosis. In contrast, STIM2 was not required for ROS production, but contributed to phagocytosis in a pathway‐dependent, collaborative manner with STIM1. This conclusion was based on a stronger decrease observed in FcγR and dectin‐1‐mediated phagocytosis in STIM1/2 neutrophils, compared to single STIM1 KO. Interestingly, neutrophil chemotaxis was found to be independent of STIM proteins both *in vitro* and *in vivo*. Responses towards fMLF and MIP‐2 in a transwell‐migration assay, as well as neutrophil recruitment in a zymosan‐induced peritonitis model were independent of both STIM1 and STIM2. Although the choice of chemokines and disease model restricts the question of an involvement of STIM proteins to neutrophil responses controlled by FPR and CXCR2‐mediated, G‐protein‐dependent signalling pathways *in vitro* and to zymosan‐mediated, TLR‐2/dectin‐1 pathway *in vivo*, these findings indicates that neutrophil migration can proceed effectively in the complete absence of STIM proteins.

Besides chemotaxis, ROS production and phagocytosis, cytokine production and release are key components in anti‐microbial responses and were therefore investigated in the study. The release of secondary granules, determined by CD11b surface marker expression and lactoferin release, was affected by STIM1, but not by STIM2 ablation. Unexpectedly, a specific requirement for STIM2, but not STIM1, was observed in the transcriptional regulation of cytokines. STIM2 was required for activation and nuclear translocation of the transcription factor NfkB, subsequently promoting the expression of the pro‐inflammatory cytokines TNFα, IL‐6, IL‐10 and CXCL‐1. *In vivo* experiments further demonstrated the physiological relevance of these findings, by showing that STIM2 and STIM1/2 double knock‐out mice are protected against acute cytokine responses caused by pathogen challenge. The protective effect was associated with the reduced cytokine production by STIM2‐ablated neutrophils in these mice. These findings highlight a new role for STIM2 in regulating cytokine production on a transcriptional level. Although this STIM2‐specific effect requires validation by other groups, an earlier study also indicated that STIM1 can be dispensable for cytokine production. Steinckwich and colleagues reported no effect of STIM1 myeloid ablation on cytokine expression triggered by IMQ‐treatment in a mouse psoriasis model (Steinckwich *et al*. 2015). Neither the expression of the key cytokines IL‐17, IL‐22 and IL‐23, nor the expression of CXCL‐1, and ‐2 was altered. Thus, two studies using two different disease models in different genetic backgrounds failed to link STIM1 to the regulation of cytokine expression, the most recent study reporting an unexpected contribution for the neglected STIM2 isoform.

The main conclusion from the Clemens study is the existence of a collaborative and differential requirement of STIM1‐ and STIM2‐mediated SOCE for distinct processes. This demonstrates the importance of a combined and comparative examination of both isoforms to uncover their distinct functions. These and other aspects are discussed in more detail in the following sections.

## Considerations in studying and interpreting the role of STIM proteins in neutrophil functions

### Choosing a model system

Studying protein functions in primary granulocytes like neutrophils is a challenging endeavour. Despite a plethora of molecular techniques, manipulating gene and protein expression remains difficult due to insufficient transfection efficiency, short cellular life span and challenging culture conditions. Without efficient strategies to manipulate protein function in primary neutrophils, only pharmacological approaches or animal models remain and in the absence of specific STIM inhibitors, mouse models are at present the best option.

#### Mouse models

STIM function in mouse models was investigated using two approaches: chimeric animals and the Cre‐lox system. Bone marrow or fetal liver chimeras provide a full ablation of STIM proteins in the haematopoietic lineage, in both the lymphoid and myeloid branches (Braun *et al*. 2009; Zhang *et al*. 2014; Sogkas *et al*. 2015). With this strategy, special attention needs to be paid during *in vivo* experiments since lymphocytes and other phagocytes deficient in STIM proteins might contribute to a phenotype. The technique uses bone marrow or fetal liver cells from knock‐out donor animals as a source for haematopoietic precursor or stem cells. These cells are transplanted intravenously to previously irradiated acceptor animals, allowing a repopulation of the system with effector cells deriving from the transplanted precursors (Duran‐Struuck & Dysko, 2009). The transplanted precursors are lost within a few months and the chimeric genotype cannot be maintained through breeding, as germ lines are unaffected by the transplantation. The Cre‐lox system can be used to introduce deletions and insertions in a gene of interest by exploiting the ability of the bacteriophage‐derived Cre recombinase to interact with two DNA recognition sites (loxP). The Cre recombinase can be introduced into genes controlled by tissue or cell type specific promotors. The loxP sites are targeted to one or several exons in the gene of interest, flanking a specific DNA sequence (referred to as ‘floxed’) Crossing desired Cre‐ and floxed‐animals will lead to a recombination, e.g. a deletion, of the floxed DNA sequence in animals carrying both components. The genetic modification is maintained through breeding and the approach provides high flexibility in promotor choice and hence the controlled tissue‐ or cell‐specific restriction of protein ablation. A disadvantage is the risk of genetic drift, caused by continued inbreeding, leading to loss of Cre activity or loxP sites. Cre‐lox models require regular back‐crossing to wild type animals and control of the genotype to confirm the introduced genetic modification, procedures not required when using a chimeric mouse model. In 2014, Abram *et al*. published a comprehensive study analysing the efficiency and suitability of different Cre‐promotors focusing on myeloid lineage restriction, providing an excellent basis for an informed decision making for choosing suitable lines and interpreting results (Abram *et al*. 2014). LysM‐, Vav1‐ and Mrp8‐Cre models have so far been used to study STIM function in neutrophils (Nunes *et al*. 2012; Steinckwich *et al*. 2015; Clemens *et al*. 2017). Vav1 is a protein involved in the early steps of haematopoietic development, allowing the promotor‐controlled activity of Cre in the myeloid and lymphoid linage. Like bone marrow chimera, the Vav1‐Cre model is not suitable for *in vivo* studies, due to difficulties in allocating observed effects to a specific immune cell type. LysM‐Cre is a commonly applied system and can be used in a hetero‐ and homozygous expression to improve protein ablation. Under the control of the lysozyme 2 promotor, Cre activity is limited to the myeloid linage with a relative late onset in granulopoiesis. This holds the risk of insufficient protein ablation. The Mrp8‐Cre model is the only neutrophil‐specific model used for STIM research so far, and the best suited for *in vivo* studies. Mrp8 encodes the S100A8 protein, a member of the large Ca^2+^‐binding S100 protein family (Steinckwich *et al*. 2011; Donato *et al*. 2013). Homozygous expression of Cre impairs Ca^2+^‐dependent neutrophil functions and is therefore not recommended. Although not used so far to study STIM function in neutrophils, CEBPα‐Cre is an interesting alternative, as CEBP proteins regulate granulopoiesis, with CEBPα acting earliest, providing a reliable activity of Cre in the myeloid lineage (Fiedler & Brunner, 2012; Burgener *et al*. 2016). As for Mrp8, homozygous Cre expression cannot be applied due to the physiological relevance of CEBPα.

#### Human cellular models

Results gained from mouse models have little therapeutic value if not replicated in humans. In the human HL‐60 cell‐line, no effect on global Ca^2+^ signals or ROS production was shown upon RNAi‐mediated STIM2 down‐regulation (Bréchard *et al*. 2009), while targeting STIM1 resulted in reduced Ca^2+^ entry, impaired cell polarization and reduced ROS production (Bréchard *et al*. 2009; Steinckwich *et al*. 2011; Zou *et al*. 2012). However, these findings were not confirmed in primary human neutrophils from a patient carrying a pR429C mutation that impairs STIM1‐ORAI1 binding (Elling *et al*. 2016). This study did not reveal a role for STIM1 in fMLP‐ or streptococci‐induced SOCE, migration towards fMLP, C5a or PAF, and phagocytosis and cytokine (IL‐8) secretion (Elling *et al*. 2016). As further discussed below, however, the study in primary neutrophils and those from HL‐60 cells differ with regard to the experimental design, functional read‐outs, and activating conditions.

#### Alternative approaches

The CRISPR‐Cas9 technique is now the method of choice for genome editing but this approach has not yet been applied to target STIM expression in neutrophils. The CRISPR‐Cas9 system is more flexible than the Cre‐lox system, since nearly every region in the gene of interest can be targeted for deletion or modification, and provides stable genomic alterations without additional factors, which facilitates breeding strategies. An alternative cellular model was introduced a decade ago by Wang *et al*. who generated an immortalized murine myeloid progenitor line that can be differentiated into primary‐like neutrophils (Wang *et al*. 2006). These cells derive from healthy animals and are kept in a proliferative, immature state by expressing the Hoxb8 gene under the control of an oestrogen promotor. Silencing Hoxb8 expression by removing oestrogen or tamoxifen from the cell culture unleashes the neutrophil differentiation programme. The technique offers advantages over other myeloid cell lines due to the non‐cancerous origin of cells and their physiological differentiation programme. Although the technique has been further developed by other groups (Gurzeler *et al*. 2013; Reinhart *et al*. 2016), no studies have been published so far using these cells to investigate SOCE. Whether these cells can serve as a reliable experimental tool to support or replace animal studies will depend on whether they can fully recapitulate the functions of terminally differentiated neutrophils. Three arguments support further exploring the technique. First, it could be applied to generate cell lines from established knock‐out mouse models, providing a source of unlimited cells for large scale experiments. Second, using lentiviral transduction, RNAi or CRISPR‐Cas9, transient or stable STIM knock‐down, or knock‐out cell lines could be generated. Third, the technique could be applied to human cells, providing alternatives to the existing myeloid cell lines, generating a new approach to confirm results from animal models in a human system.

### Isoform choice and experimental design

Neutrophils express both STIM1 and STIM2 proteins, but the relative expression of the two isoforms is not firmly established and is likely to vary during maturation and differentiation and following priming or pathogen exposure (Clemens *et al*. 2017). Quantitative proteomic and transcriptional profiles are needed to document the presence of the different STIM isoforms and splice variants in resting and activated neutrophils. STIM1 and STIM2 have different Ca^2+^‐ and lipid‐binding affinities, consistent with a differential use of STIM isoforms in various Ca^2+^‐dependent functions (Brandman *et al*. 2007; Ercan *et al*. 2009). STIM2 is activated by mild ER Ca^2+^‐store depletion and was initially proposed to regulate basal Ca^2+^ homeostasis or to act as a STIM1 inhibitor (Soboloff *et al*. 2006; Brandman *et al*. 2007). Recent studies, however, showed that STIM2 actively regulates SOCE in neurons, melanoma or monocytes (Berna‐Erro *et al*. 2009; Stanisz *et al*. 2014; Saul *et al*. 2016). The presence of two isoforms activated by different conditions increases the diversity of cellular responses towards pathogen‐ and host‐associated ligands, cytokines, and paracrine signals, a versatility enabling neutrophils to respond to a broad range of stimuli.

The versatility in generating Ca^2+^ signals driving specific cellular responses is not only provided by differentially regulated STIM molecules, but also by their ability to interact with different types of channels. Neutrophils express a plethora of TRP channel family members, such as TRPV5, TRPV6, TRPM2 and TRPC1, that enable permeation of Ca^2+^ (Gees *et al*. 2010) and thus control Ca^2+^ dependent functions. For instance, TRPC1 was shown to mediate migration and chemotaxis (Lindemann *et al*. 2015). STIM molecules were shown to bind and gate not only ORAI channels, but also TRPC1, and the cooperative action of TRPC, ORAI and STIM molecules in mediating SOCE is of growing interest. We refer readers to the relevant reviews (Saul *et al*. 2013; Bodnar *et al*. 2017; Ong & Ambudkar, 2017). To understand how STIM proteins regulate diverse neutrophil functions, it will be necessary to identify the ORAI and/or TRPC channel(s) interacting with the different STIM isoforms following activation by different stimuli and to link these distinct Ca^2+^ entry pathways to different downstream signalling processes. Given the paucity of neutrophil studies analysing simultaneously the role of STIM proteins and of their target channels, the following paragraphs will focus on the STIM1 and STIM2 proteins.

**Figure 1:**
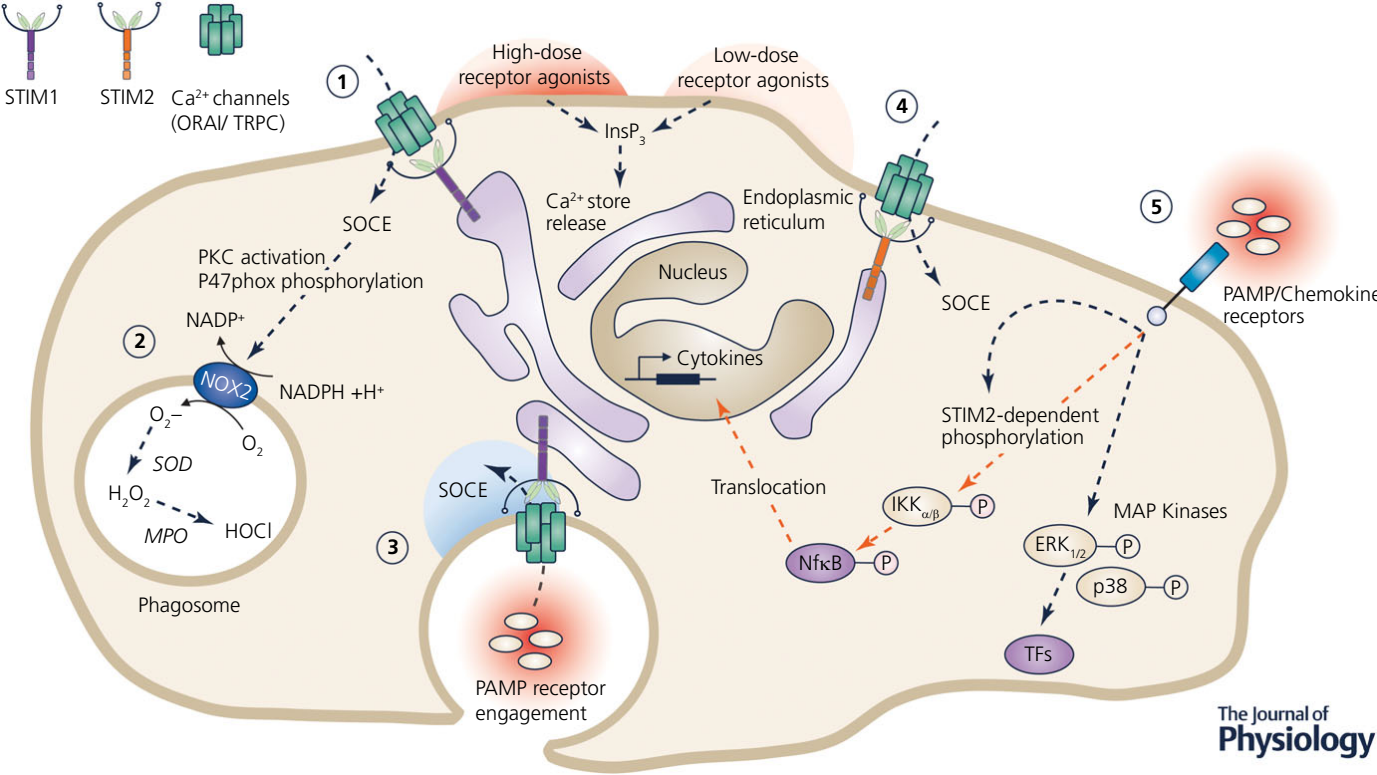
Established and suggested STIM1‐ and STIM2‐regulated neutrophil functions (1) Store‐operated Ca^2+^ entry (SOCE) mediated by STIM1‐ORAI1 is triggered by high‐dose receptor stimulation, for example FPRs, PAFR or C5aR. Receptor engagement results in InsP_3_ generation, binding of its cognate receptor in the endoplasmic reticulum (ER) and the release of Ca^2+^ from ER‐Ca^2+^ stores. This initiates STIM1 conformational change, translocation and gating of plasma‐membrane residing channels such as ORAI or TRPC. (2) SOCE contributes to the assembly and stabilization of the NADPH oxidase (NOX2), e.g. by activating PKC, in collaboration with additional factors such as DAG, to provide phosphorylation of the cytosolic NOX2 subunit p47^phox^. NOX2 produces superoxide (O_2_^−^) anions that in the phagosome are enzymatically transformed to hydrogen peroxide (H_2_O_2_) and hypochlorous acid (HOCl) by superoxide dismutase (SOD) and myeloperoxidase (MPO), respectively. (3) Phagocytosis, mediated by engagement of pathogen‐associated molecular patterns (PAMP) with specific receptors, is dependent on SOCE. (Suggested) Phagocytosis is promoted by local Ca^2+^ signals fuelled by STIM1‐ORAI1 interactions on phagosomes. (4) Low‐dose agonist receptor engagement, for example FPR activation by low doses (<100 nM) of its agonist fMLF, activates SOCE mediated by STIM2. (5) (Suggested) STIM2‐mediated SOCE is triggered by PAMP/Chemokine‐receptor engagement and involved in the Ca^2+^‐dependent phosphorylation and activation of downstream signalling cascades activating IKKα/β kinase and the transcription factor (TF, purple) NfkB, regulating the expression of target genes (e.g. cytokine expression). The MAP‐kinase pathway and the phosphorylation of kinases p38 and ERK_1/2_ were shown not to be regulated by STIM2. Dashed lines indicate multi‐step interactions, orange lines indicate STIM2 dependency, P indicates phosphorylation. Only the functions and molecular players linked to STIM‐mediated Ca^2+^ signalling are depicted, excluding a broad range of factors contributing to ROS production, phagocytosis and cytokine synthesis. Abbreviations: MAP, mitogen‐activated protein (kinase); IKK_α/β,_ IκB kinase alpha/beta; ERK, extracellular signal‐regulated kinase; NfkB, nuclear factor ‘kappa‐light‐chain‐enhancer’ of activated B‐cells; InsP_3_, inositol trisphosphate; FPR, formylated peptide receptor; PKC, protein kinase C.

Whether specific neutrophil functions are predominantly regulated by STIM1 or STIM2 is still not firmly established, since only a few studies have analysed the function of the two proteins in parallel. As summarized by Nunes and Demaurex (Demaurex & Nunes, 2016), five studies covered solely STIM1 in animal models (Braun *et al*. 2009; Nunes *et al*. 2012; Zhang *et al*. 2014; Sogkas *et al*. 2015; Steinckwich *et al*. 2015), while three studies addressed STIM1 using an RNAi approach in HL‐60 cells (Bréchard *et al*. 2009; Steinckwich *et al*. 2011; Zou *et al*. 2012). Elling *et al*. provided the only study using human primary cells derived from patients (Elling *et al*. 2016) and a single study addressed STIM1 function in a mouse model, as well as in human HL‐60 cells (Steinckwich *et al*. 2015). Two studies investigated STIM2, one using RNAi in HL‐60 cells (Bréchard *et al*. 2009), and the other study by Clemens *et al*. is the only one investigating simultaneously the role of the two isoforms in mouse neutrophils (Clemens *et al*. 2017). To date, studies focusing on the role of STIM2 are clearly underrepresented. Nevertheless, a tendency is emerging to link STIM1 to major neutrophil functions. Figure 1 provides a simplified summary of functions reported to be, at least partially, dependent on STIM1‐ or STIM2‐mediated Ca^2+^ signalling. Several studies consistently reported that global Ca^2+^ signals and Ca^2+^‐dependent ROS production require STIM1 (Bréchard *et al*. 2009; Steinckwich *et al*. 2011; Zhang *et al*. 2014). In contrast, STIM2 appears dispensable for global Ca^2+^ signals and ROS production and is only required under specific conditions. As reported by Clemens *et al*., FPRs differentially engage STIM1 or STIM2 depending on the agonist concentration, with a low dose (<100 nm) of bacterial‐derived fMLF activating STIM2, and high doses preferentially activating STIM1 (Clemens *et al*. 2017). This is supported by an early study that revealed an agonist‐dependent activation of STIM1 and STIM2 (Kar *et al*. 2012). Additional studies are required to confirm reports of STIM1 and STIM2 providing local Ca^2+^ signals, as so far only one study has reported a role for STIM1 in sustaining Ca^2+^ hotspots during phagocytosis (Nunes *et al*. 2012). Degranulation and cytokine production and secretion are crucial factors for pathogen clearance, tissue remodelling and signalling and neutrophils use a uniquely broad variety of granules and cytokines. The implication of STIM proteins in controlling this complex array of neutrophil responses is still incomplete. Three out of five studies investigating cytokine release focused solely on STIM1 and restricted their focus to secretion of single cytokines, either IL‐8 (Elling *et al*. 2016), TNFα, or CXCL‐2 (Braun *et al*. 2009; Sogkas *et al*. 2015), reporting the secretion to be either decreased or not affected by STIM1 ablation. Steinckwich *et al*. performed a more detailed analysis by investigating cytokine synthesis on a transcriptional level, but also focused only on STIM1. They reported that the transcription of the key cytokines mediating the inflammatory response (IL‐17, IL‐22, IL‐23 and CXCL‐1 and ‐2) was not altered by STIM1 ablation in their psoriasis model. Only Clemens and colleagues, in the most comprehensive analysis so far, investigated cytokine synthesis, production and release, and also considered a role of STIM2 in this crucial process.

**Figure 2:**
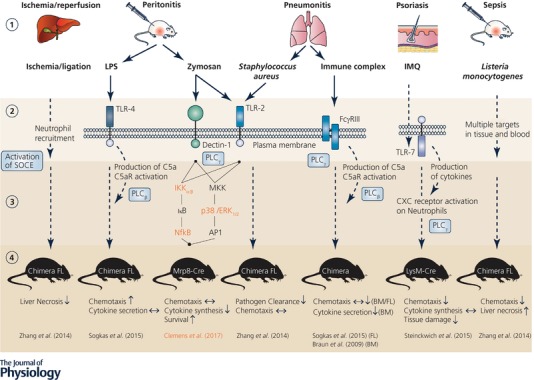
Diseases, pathways, and targets investigated in mice models of STIM1/2 myeloid ablation Summary of the different layers of comparing *in vivo* mouse models investigating STIM1 and STIM2 (indicated in orange) knock‐outs. (1) Disease models. Indicated are the investigated disease models, primary affected organs and applied challenges. (2) Molecular targets. Challenges can activate neutrophils either directly (arrows) or indirectly (dashed lines). Potential primary receptors targeted are: Toll‐like receptor (TLR)‐4 for LPS, TLR‐2 and dectin‐1 for zymosan and *Staphylococcus aureus* and Fcγ receptor III (FcγRIII) for immune complexes (non‐exclusive selection). Indirect activation of neutrophils occurs in the ischaemia model, where neutrophils are recruited to the site of vessel ligation, the sepsis model, where a broad range of cells is affected, and the imiquimod (IMQ) psoriasis model, where the primary target is TLR‐7‐mediated production of cytokines by dendritic cells and macrophages, that further activate e.g. CXC‐chemokine receptors on neutrophils. (3) Downstream signalling. Most studies did not investigate the downstream pathways affected by STIM1 KO in the disease model. Possible final targets of the engaged receptors include the transcription factors NfKB and AP‐1, involved in pro‐inflammatory gene expression. *C*omponents affected by the knock‐out of STIM2 are indicated in orange. Phosphorylation of MAP‐kinases p38 and ERK_1/2_ was not affected by STIM2 ablation, while phosphorylation of the kinase IKK_α/β_ and the transcription factor NfκB was reduced in STIM2 and STIM1/2 knock‐outs. Phospholipases (PLC) mediating SOCE activation via InsP_3_ generation and ER Ca^2+^ store release are indicated in blue. (4) Animal models and phenotype. Animal models used are: fetal liver chimeras (FL), bone‐marrow chimeras (BM), Mrp8‐Cre‐lox (Mrp8) and LysM‐Cre‐lox (LysM). The effects of STIM1 (STIM2 in orange) ablation on the investigated functions and features are indicated by: ↑, increased; ↓, decreased; ↔ not altered. Abbreviations: LPS, lipopolysaccharide; IKK_α/β_, IκB kinase alpha/beta; MKK, mitogen‐activated protein kinase kinase; ERK, extracellular signal‐regulated kinase; NfkB, nuclear factor ‘kappa‐light‐chain‐enhancer’ of activated B‐cells; AP‐1, activator protein 1.

Lack of standardisation also prevents firm conclusions on the role of STIM proteins in neutrophils. An obvious example is chemotaxis where STIM1 implication was repeatedly tested using different settings. As depicted in Fig. 2 and discussed in detail in the following section, the use of different model systems, variations in experimental conditions, and differences in the signalling pathways engaged by different agonists all complicate a comparison of the results. Figure 2 summarizes the *in vivo* studies performed on STIM1 and STIM2 knock‐out animal models. The receptors and downstream signalling pathways depicted are the most likely molecular targets based on the information provided by the individual studies but do not provide an exhaustive description, as other so‐far‐unidentified molecular players may also be involved. Among five *in vivo* studies, three studies failed to reveal a role for STIM1 in neutrophil recruitment in either a zymosan‐induced peritonitis (TLR‐2 signalling dependent), a *S. aureus‐*induced pneumonitis (among others TLR‐2 signalling dependent*)*, and an immune complex (IC)‐induced pneumonitis (FcyR signalling dependent), using a Mrp8‐Cre‐lox (Clemens *et al*. 2017), a fetal liver chimera (Zhang *et al*. 2014) or a bone marrow chimera model (Braun *et al*. 2009), respectively. In the pneumonitis study using *S.aureus*, however, an increased bacterial burden was related to STIM1 ablation, indicating a defect in myeloid (and lymphoid) cell‐dependent pathogen clearance (Zhang *et al*. 2014). The same study also reported this effect of STIM1 ablation in a *Listeria monocytogenes*‐induced sepsis and the resulting bacterial burden in liver and spleen. The remaining two studies did report an effect of STIM1 ablation on neutrophil chemotaxis or recruitment, though with contrasting results. Sogkas *et al*. investigated a lipopolysaccharide (LPS)‐induced peritonitis (TLR‐4 signalling dependent) and an IC‐induced pneumonitis (FcyR signalling dependent) in a fetal liver chimeric model. The different disease models revealed opposite effects of STIM1 ablation, with neutrophil recruitment being increased in the peritoneal cavity and decreased in the lung (Sogkas *et al*. 2015). Steinckwich et al. investigated an Imiquimod‐induced (TLR‐7 signalling dependent) psoriasis and reported a reduced neutrophil recruitment to infected skin lesions and decreased associated tissue damage in a LysM‐Cre model (Steinckwich *et al*. 2015). *In vitro* studies were more consistent since all used transwell‐migration assays, the majority reporting no involvement of STIM1 in chemotaxis. A diverging study reported a role for STIM1 but used additional chemoattractants not tested by other groups (Steinckwich *et al*. 2015). The majority of studies focused on classical chemoattractants such as fMLF and MIP‐2 (CXCL‐2). Steinckwich and colleagues also tested migration towards fMIVIL (low dose), WKYMVM (peptide) and KC (CXCL‐1). While all chemokines tested in transwell assays targeted either FPR (fMLF, fMIVIL, WKYMVM) or CXCR2 (MIP‐2, KC) to induce chemotaxis, it is not clear whether differences in the mouse models, or in the chemokine doses might result in a differential activation of specific downstream pathways and subsequently distinct final cellular responses.

A detailed analysis of downstream targets is crucial to reveal pathways specifically controlled by STIM1 and STIM2 and to clarify their redundancies or synergies, as recently shown by Clemens and colleagues. Most *in vitro* and *in vivo* tested stimuli target G‐Protein‐coupled receptors (GPRCs) (such as FPR, CXCR2, C5aR), FcyRs or TLRs. Although the range of receptors is limited, the downstream signalling cascades activated are multiple and difficult to predict, involving different adapter proteins for TLRs, phospholipases (PLC) for GPCRs and FcyRs, and different kinases (IKK, MAPK) for FcyRs, TLRs and GPCRs. How STIM1‐ and STIM2‐mediated SOCE differentially triggers these, and potentially other, signalling pathways in neutrophils, thereby engaging the activation of specific functions or transcription factors, is still unclear. Clemens *et al*. demonstrated that specific targets can be identified, but the molecular details of the underlying cellular signalling circuit remain to be clarified. Without a detailed analysis of the downstream components that are activated, it is difficult to associate the observed effects of STIM ablation to specific molecular targets. Further studies are required to understand how separate or simultaneous activation of STIM1 and STIM2 can activate specific signalling cascades.

Taken together, the described differences in experimental design call for future replication studies that should cover a broad range of stimuli targeting different sub‐cellular pathways and use comparable experimental set‐ups *in vitro* and *in vivo*. Standardization procedures aiming to minimize system‐ and design‐related differences should be adopted to clarify the role of STIM1 and STIM2 proteins, not only in chemotaxis but also other neutrophil functions.

## Closing remarks

The excellent work of several groups has provided significant insight into the regulation of SOCE‐dependent functions by STIM proteins in neutrophils. Nonetheless, there is a strong need for replication studies to confirm the dominant role of STIM1 in ROS production, to clarify diverging reports linking STIM1 to impaired or enhanced chemotaxis and neutrophil recruitment and to elucidate the involvement of the two isoforms in degranulation and cytokine secretion. An important and overlooked aspect will be to improve the standardization of experimental procedures to allow proper comparison of future studies. Due to the specific focus of this short review we were not able to include all publications concerning the role of SOCE in neutrophil function and would like to apologize to those colleagues whose work we could not include.

## Additional information

### Competing interests

The authors declare no competing financial interests.

### Author contributions

N.D. and S.S. outlined and wrote the review. N.D. edited the final version of the manuscript. S.S. designed the figures. Both authors accept the final version of the manuscript as submitted.

### Funding

This work was funded by grant 31003A‐149566 and CRSII3_ 160782 of the Swiss national science foundation (SNF) awarded to N.D.
